# “一锅法”制备氨基碳纳米管功能化磁性纳米粒子及其在谷物和蔬菜中苯氧羧酸类除草剂测定中的应用

**DOI:** 10.3724/SP.J.1123.2021.12008

**Published:** 2022-10-08

**Authors:** Youfang HUANG, Jun LIU, Xiaojia HUANG

**Affiliations:** 厦门大学环境与生态学院, 福建 厦门 361000; College of the Environment and Ecology, Xiamen University, Xiamen 361000, China

**Keywords:** 氨基化碳纳米管, 磁固相萃取, 高效液相色谱, 苯氧羧酸, 除草剂, 谷物, 蔬菜, aminated carbon nanotubes, magnetic solid-phase extraction (MSPE), high performance liquid chromatography (HPLC), phenoxyacetic acid, herbicides, cereals, vegetables

## Abstract

有效萃取是分析复杂样品中苯氧羧酸类除草剂(PAs)残留的关键步骤。为此,该文利用“一锅法”水热技术快速、简便地制备了氨基碳纳米管功能化磁性纳米粒子(NH_2_-CNTs@M)并作为磁固相萃取(MSPE)的萃取介质,用于萃取谷物和蔬菜样品中痕量PAs。研究利用多种手段对NH_2_-CNTs@M的形貌、尺寸、磁性性质等进行了表征,结果表明Fe_3_O_4_的粒径、氨基化碳纳米管的直径以及NH_2_-CNTs@M的磁饱和值分别为30 nm、40 nm和44.2 emu/g。详细考察了制备条件和萃取参数对NH_2_-CNTs@M/MSPE萃取性能的影响,结果表明,NH_2_-CNTs@M/MSPE可通过*π-π*、疏水和氢键作用有效富集目标化合物,最佳萃取条件如下:吸附剂用量为30 mg,解吸溶剂为含2.0%(v/v)甲酸的乙腈溶液,吸附时间和解吸时间分别为8.0 min和3.0 min,基底pH值为6.0,不调节基底的离子强度。将NH_2_-CNTs@M/MSPE与高效液相色谱-二极管阵列检测技术(HPLC-DAD)联用,建立了谷物和蔬菜中PAs的灵敏检测方法。谷物和蔬菜基质中苯氧羧酸类除草剂的检出限(LOD, *S/N*=3)分别为0.32~1.6 μg/kg和0.53~1.6 μg/kg,定量限(LOQ, *S/N*=10)分别为0.94~4.8 μg/kg和1.6~4.8 μg/kg。在两种实际样品中不同浓度下的加标回收率分别为73.1%~112%和72.3%~113%。与现有方法相比,所建方法具有萃取速度快、灵敏度高和环境友好等特点。

## Abstract (en)

Phenoxyacetic acid herbicides (PAs) are widely used to control the growth of broad-leaf weeds in corn, tobacco, etc. The presence of PAs in plants even at low concentrations (at the ng/L to μg/L scale) may induce severe effects and lead to human health risks. Hence, a sensitive and reliable method for the determination of PAs at trace levels in cereals and vegetables is highly desired. Magnetic solid-phase extraction (MSPE) has attracted considerable attention on account of its benefits such as ease of separation, less solvent consumption, and good service life. In this study, aminated carbon nanotube-modified magnetic nanoparticles (NH_2_-CNTs@M) were prepared by a convenient and simple “one-pot” strategy and employed as the adsorbent for the MSPE of PAs in crops. The fabrication procedure is very convenient. In detail, the aminated carbon nanotubes, Fe(Ⅱ), Fe(Ⅲ), and isopropanol were mixed in one pot with mechanical stirring and reacted for 2.0 h at 80 ℃. The spectroscopic properties, morphology, and magnetic properties of the synthetic adsorbent were characterized by Fourier Transform-infrared spectroscopy (FT-IR), scanning electron microscopy (SEM), and transmission electron microscopy (TEM). The results revealed that the size of Fe_3_O_4_, diameter of NH_2_-CNTs, and the magnetic saturation values of NH_2_-CNTs@M were 30 nm, 40 nm, and 44.2 emu/g, respectively. Additionally, the results of FT-IR and TEM characterization confirmed the successful fabrication of NH_2_-CNTs@M by this “one-pot” hydrothermal approach. The NH_2_-CNTs@M displayed satisfactory capability to capture PAs through *π-π*, hydrophobic, and hydrogen-bonding interactions. To realize the best extraction performance, the key parameters, including the amount of adsorbent, formic acid concentration in the eluent, adsorption and desorption time, sample pH, and ionic strength in the sample matrices, were inspected and studied in detail. The optimal conditions were as follows: amount of NH_2_-CNTs@M, 30 mg; desorption solvent, 0.5 mL acetonitrile containing 2.0% (v/v) formic acid; adsorption and desorption times, 8.0 and 3.0 min, respectively; the sample pH was adjusted to 6.0, and no salt was added to the sample. Under the optimized extraction conditions, a sensitive, quick, and environmentally friendly method for the determination of the studied PAs in cereal and vegetable samples was established by the combination of NH_2_-CNTs@M/MSPE with high performance liquid chromatography-diode array detection (HPLC-DAD). The enrichment factors for the studied PAs varied from 73 to 90. The limits of detection (*S/N*=3) for the PAs in the cereal and vegetable matrixes were in the ranges of 0.32-1.6 μg/kg and 0.53-1.6 μg/kg, respectively; and the limits of quantification (*S/N*=10) for the PAs in the cereal and vegetable matrixes were 0.94-4.8 μg/kg and 1.6-4.8 μg/kg. The developed method also showed wide linear ranges and good precision. Finally, the established NH_2_-CNTs@M/MSPE-HPLC-DAD approach was applied to measure trace levels of PAs in cereals and vegetables, and good fortified recoveries (72.3% to 113%) and repeatability (RSDs below 10%) were obtained. The established approach has several advantages over the existing methods, such as high analytical speed, low LODs, and eco-friendliness.

苯氧羧酸类除草剂(phenoxyacetic acid herbicides, PAs)是一类开发较早的农药,由于具有除草效率高、用量小、价格低等特点,目前在水稻、小麦和玉米等农作物耕地中广泛使用^[1,2]^。因其具有强的水溶性、极性和稳定性,可以进入植物体循环系统内,参与生态循环,最终通过食物链进入人体^[3]^。研究表明,PAs及其代谢产物具有潜在毒性和致癌性,因此,PAs已被列入欧洲优先控制污染物名录中^[4]^。目前许多国家和地区规定了农作物中PAs的最高残留量(MRLs),例如欧盟((EC)No 396/2005)规定谷类和蔬菜中2,4-二氯苯氧乙酸(2,4-dichlorophenoxyacetic acid, 2,4-D)和2-甲基-4-氯苯氧乙酸(2-methyl-4-chlorophenoxyacetic acid, MCPA)的MRLs为0.05 mg/kg^[5]^。我国农业部发布的《食品安全国家标准 食品中农药最大残留限量》(GB 2763-2021)规定2,4-D和MCPA在谷物和蔬菜中的MRLs分别为0.01~2 mg/kg和0.05~0.2 mg/kg^[6]^。基于实际需求,发展可用于谷物和蔬菜中PAs含量测定的有效、灵敏和可靠的检测方法显得尤为重要。

目前,用于PAs检测的技术主要为气相色谱(GC)^[7]^、毛细管电泳(CE)^[8]^和高效液相色谱(HPLC)^[9,10]^。其中,利用GC测定PAs时,需要复杂的衍生化过程^[7]^。HPLC相较于GC不需要进行衍生化处理,并且比CE具有更高的灵敏度和灵活性,因此HPLC被广泛地应用于PAs的分析测定。由于谷物和蔬菜中PAs的含量低,且基底干扰大,难以直接进行HPLC测定,分析检测前需进行必要的样品前处理。迄今为止,已有诸多样品前处理方法用于富集复杂基底中的PAs,如固相萃取(SPE)^[10,11]^、分散固相微萃取(D-μ-SPE)^[12]^、针尖固相萃取(PT-SPE)^[13]^、纤维束固相微萃取(FB-SPME)^[2,9]^、分散液-液微萃取(DLLME)^[7]^、混合基质膜萃取(MMM)^[14]^和磁固相萃取(MSPE)^[4,15]^。其中MSPE由于具有萃取速度快、操作简便、易回收、低成本和有机溶剂使用量少等优点而受到人们的青睐^[16]^。在MSPE中,具有高萃取性能的磁性吸附材料是核心,目前虽已有例如碳质材料^[3,4,15]^、金属有机骨架^[11,13,14]^、离子液体^[17]^、有机硅材料^[8]^、分子印迹聚合物^[18,19]^等功能化的磁性纳米材料报道,但其制备过程需要多步的合成步骤,且对PAs的萃取性能有待于提高。因此发展基于快速、简便的“一锅法”水热技术制备对PAs具有良好萃取性能的功能化磁性纳米粒子对于复杂基底中PAs残留检测具有重要的现实意义。

碳纳米管(CNTs)是由石墨烯片卷成的单层或多层的管状结构材料,作为吸附剂已成功地应用于各种有机污染物的萃取^[19]^。碳纳米管因其具有亲脂性以及非极性共价键结构而呈现疏水性,在水中的分散性较差,易于聚集,因此需在其表面引入亲水性官能团,改善其分散性的同时还可以提高对有机物的萃取性能^[20]^,如引入亲水官能团(-COOH、-OH等)的CNTs可以通过氢键、*π-π*和静电相互作用等提高对具有极性基团的有机物的吸附性能^[21]^。基于功能化CNTs的优点,本实验通过“一锅法”水热制备技术快速合成了氨基碳纳米管功能化的磁性纳米粒子(NH_2_-CNTs@M),利用扫描电镜、透射电镜、红外光谱等手段对制备的纳米粒子形貌进行表征,并对吸附剂用量、吸附解吸时间、解吸溶剂、样品pH等萃取条件进行优化。在最佳萃取条件下与HPLC联用,建立对谷物和蔬菜中5种PAs的高灵敏检测方法并用于实际样品分析。

## 1 实验部分

### 1.1 仪器与试剂

高效液相色谱仪(日本Shimadzu公司):配备LC-20AB二元输液泵,SPD-M20A型DAD检测器和SIL-20A自动进样器;IEISS SUPRA 55型扫描电子显微镜(SEM,德国); JEM-1400型透射电子显微镜(TEM,日本); Avatar-360型傅里叶变换红外光谱仪(FT-IR,日本Shimadzu公司); PPMS-9型振动样品磁力计(VSM,美国Quantom公司); PE 2400型元素分析仪(VSN,美国); SHZ-82型恒温振荡器(常州国华仪器有限公司)。

PAs标准品:苯氧乙酸(phenoxyacetic acid, POA,纯度大于98%)、4-氯-苯氧乙酸(4-chlorophenoxyacetic acid, CPOA,纯度大于98%)、2,4-D(纯度大于97%)、2-硝基苯氧基乙酸(2-nitrophenoxyacetic acid, NPOA,纯度大于98%)、MCPA(纯度99.7%)均购自梯希爱(上海)化成工业发展有限公司;FeCl_3_·6H_2_O、FeCl_2_·4H_2_O、异丙醇、乙二胺、甲酸(formic acid, FA)和NaCl均购自广州西陇化工有限公司;氨基化碳纳米管(NH_2_-CNTs)购自南京先丰材料科技有限公司;乙腈(acetonitrile, ACN)、乙醇(ethanol, EtOH)和甲醇(methanol, MeOH)均为色谱纯(美国Fisher公司);超纯水由Milli-Q纯水系统(美国Millipore公司)制得。

标准溶液配制:称取5种PAs标准品各10 mg,分别用MeOH定容至10 mL,配成质量浓度为1000 mg/L的单标储备液;各取1.0 mL的单标储备液,用MeOH定容至10 mL,配成100 mg/L的混合标准使用液。所有溶液均置于4 ℃的冰箱中备用。

### 1.2 色谱条件

色谱柱为Thermo BDS Hypersil C18柱(250 mm×4.6 mm, 5.0 μm);流动相:0.1%(v/v)磷酸水溶液(A)和ACN-MeOH(2∶3, v/v)(B);采用等度洗脱,流动相为A-B(55∶45, v/v);流速:1.0 mL/min;进样体积:20 μL;检测波长:200 nm。

### 1.3 NH_2_-CNTs@M的制备

称取270 mg FeCl_3_·6H_2_O、100 mg FeCl_2_·4H_2_O和30 mg NH_2_-CNTs分散在100 mL异丙醇-水(1∶1, v/v)溶液中,超声使溶液分散均匀,转移至三口烧瓶置于水浴锅中,加热搅拌升温至60 ℃,逐滴加入10 mL乙二胺,在氮气保护下升温至80 ℃,继续搅拌反应2 h。反应完成后,将得到的纳米粒子用MeOH和水反复洗涤,置于70 ℃的烘箱中烘干4 h即可得到NH_2_-CNTs@M。在不添加NH_2_-CNTs的情况下,根据相同的方法制备了Fe_3_O_4_纳米粒子。NH_2_-CNTs@M的制备流程见图1。

**图1:**
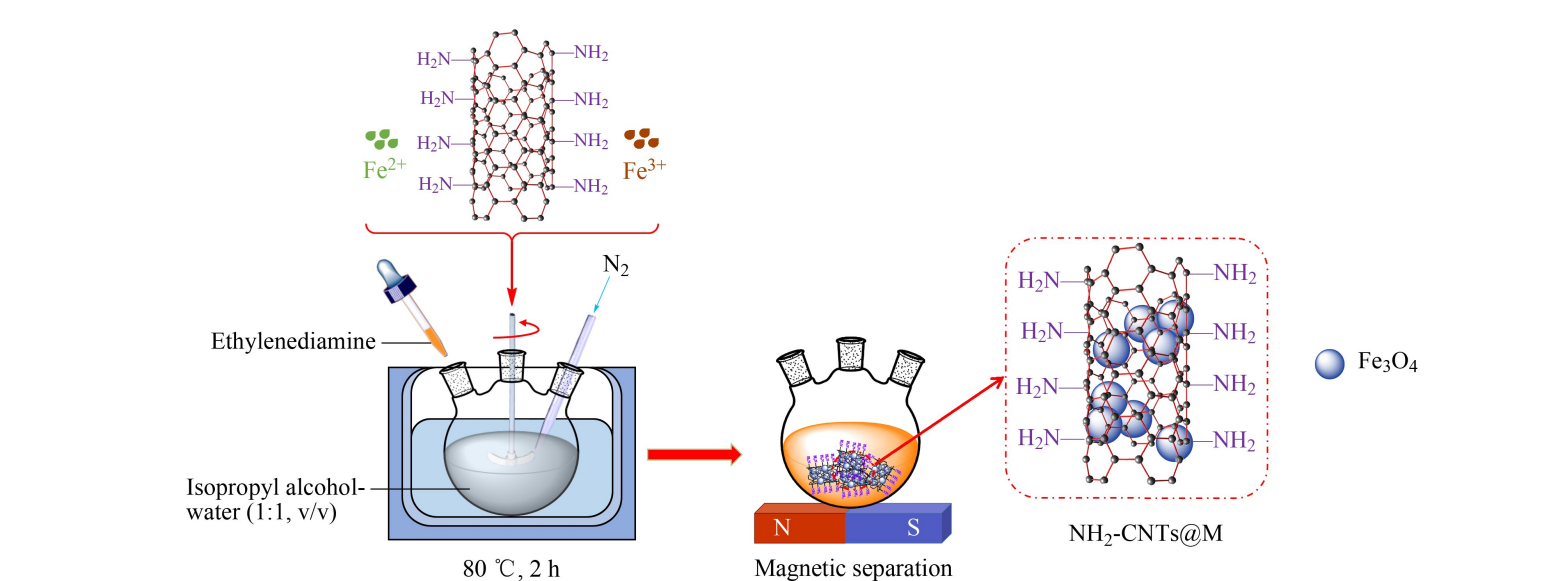
NH_2_-CNTs@M的制备流程图

### 1.4 NH_2_-CNTs@M/MSPE萃取过程

准确称取30 mg的磁性碳纳米管置于100 mL的离心管中,用甲醇和水分别活化后,加入50 mL样品溶液(pH=6,不调节离子强度),在室温下振荡(230 r/min)吸附8.0 min,吸附完成后,在外置磁场作用下将吸附剂与试样分离。倒去废液,然后加入0.5 mL的含2.0%(v/v) FA的乙腈,在130 r/min转速下解吸3.0 min。解吸液用0.22 μm的聚四氟乙烯(PTFE)滤膜过滤后待测。使用后的NH_2_-CNTs@M用含2.0%(v/v) FA的乙腈去除残留,然后用甲醇、水各浸泡5 min,烘干即可用于萃取其他样品。

### 1.5 实际样品预处理

谷物样品大米、小米和面粉分别采自山西、青海和黑龙江;蔬菜样品(黄瓜和青瓜)均采购自当地超市。

谷物样品处理:谷物样品经粉碎机磨碎后过40目筛,称取过筛后的谷物1.0 g于10 mL的离心管中,加入2.0 mL的含2.0% (v/v) FA的乙腈提取液,均质后,超声提取15 min,在3500 r/min的离心机中离心15 min,取上清液;重复上述提取操作,将上清液置于吹托管中并氮吹至干,然后用超纯水定容至50 mL, 0.1 mol/L HCl调节样品溶液pH值为6.0,不调节离子强度,然后利用NH_2_-CNTs@M/MSPE进行萃取。

蔬菜样品的处理过程:蔬菜样品用榨汁机搅碎后,称取2.0 g样品于10 mL的离心管中,提取步骤与谷类样品处理相同,在合并的上清液中加入过量的无水硫酸钠除水后将其氮吹至干,加入50 mL超纯水并调节样品溶液pH至6.0,然后进行NH_2_-CNTs@M/MSPE萃取。

## 2 结果与讨论

### 2.1 NH_2_-CNTs@M用量的优化

由于吸附剂主要通过PAs与NH_2_-CNTs之间的疏水、*π-π*、氢键及偶极-偶极作用,而NH_2_-CNTs含量影响着吸附剂官能团的丰度,从而影响和目标物之间作用力的大小及萃取性能,因此需要对合成过程中NH_2_-CNTs用量进行考察。本研究考察的NH_2_-CNTs用量范围为10~50 mg,结果如图2所示。

**图2:**
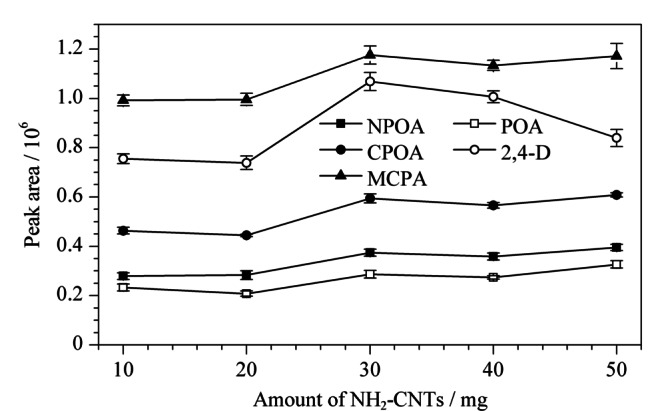
NH_2_-CNTs的用量对5种目标物萃取性能的影响

由图2可见,NH_2_-CNTs用量从10 mg增加至30 mg时,NH_2_-CNTs@M对目标物的吸附效果逐渐增加,而从30 mg到50 mg则吸附量未有明显提高,因此选择30 mg为最佳NH_2_-CNTs用量。在最佳的NH_2_-CNTs@M用量条件下,考察NH_2_-CNTs@M的制备重复性,不同批次合成的磁性吸附剂对PAs萃取性能的RSD值(*n*=4)为6.37%~9.75%。同时,实验结果表明,制备的NH_2_-CNTs@M可重复使用50次以上,具有良好的稳定性和使用寿命。

### 2.2 NH_2_-CNTs@M的表征

NH_2_-CNTs@M的FT-IR结果如图3a所示。通过比较NH_2_-CNTs、Fe_3_O_4_和NH_2_-CNTs@M的红外光谱图可以发现,NH_2_-CNTs@M谱图上存在NH_2_-CNTs和Fe_3_O_4_的主要吸收峰,如1637 cm^-1^和1399 cm^-1^处的吸收峰为氨基基团的伸缩振动峰,在571 cm^-1^处的吸收峰表明Fe-O键的存在。

**图3:**
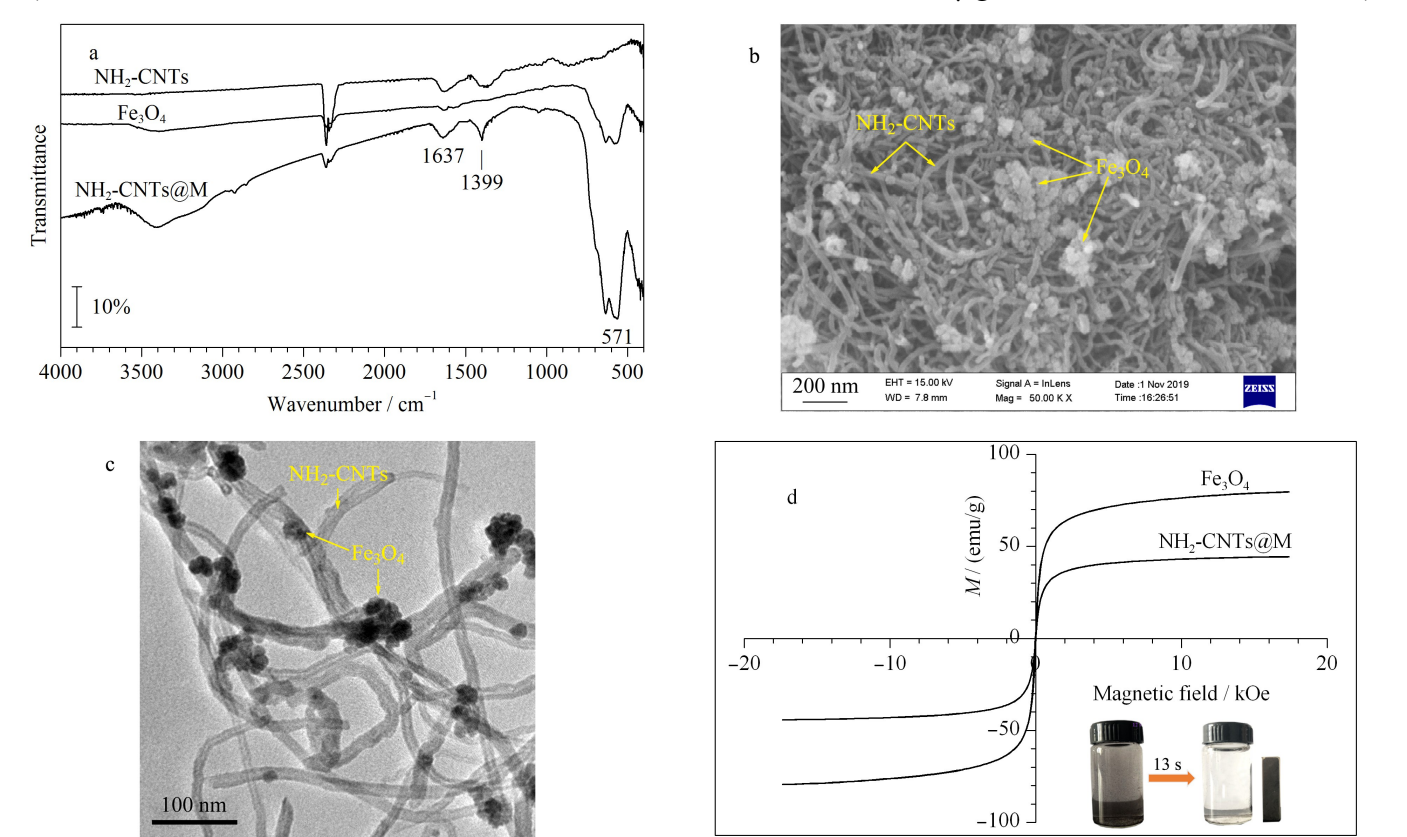
NH_2_-CNTs@M的(a)红外光谱图、(b)扫描电镜图、(c)透射电镜图和(d)磁化曲线

FT-IR结果表明NH_2_-CNTs成功修饰了Fe_3_O_4_粒子。图3b和图3c分别为NH_2_-CNTs@M的SEM图和TEM图。从SEM图中可以直观地观察到Fe_3_O_4_粒子和NH_2_-CNTs。Fe_3_O_4_的粒径和NH_2_-CNTs的直径可从TEM图进行测算,其值分别为30 nm和40 nm,并且可以清楚地观察到Fe_3_O_4_和NH_2_-CNTs相互交结一起。图3d为NH_2_-CNTs@M和Fe_3_O_4_在室温下的磁化曲线,图中显示NH_2_-CNTs@M具有典型的顺磁性,由于NH_2_-CNTs缠绕着Fe_3_O_4_使得NH_2_-CNTs@M的磁饱和值(MSV)有所降低(MS $V_{NH_{2}-CNTs@M}$ =44.2 emu/g, MS $V_{Fe_{3}O_{4}}$ =79.4 emu/g),但是分散的磁性粒子仍可在13 s内在外置磁力作用下快速聚集而与样品溶液分离。上述表征结果均表明利用“一锅法”成功制备了NH_2_-CNTs@M,且制备得到的纳米材料具有良好的磁化强度和顺磁性。

### 2.3 萃取条件优化

为了取得最佳萃取性能,本研究对可能影响NH_2_-CNTs@M/MSPE萃取性能的一系列参数进行了优化,包括吸附剂用量、吸附和解吸时间、解吸溶剂、基底的pH和盐度。优化过程是在50 mL的超纯水中加入100 μg/L的PAs混合标准使用液,并通过色谱峰的峰面积评估NH_2_-CNTs@M/MSPE对PAs的萃取性能。

#### 2.3.1 吸附剂用量

吸附剂用量的考察范围为10~50 mg,结果如图4所示。可以看出,NH_2_-CNTs@M对PAs的萃取性能随着吸附剂用量的增加而增强,并在30 mg时达到最佳效果,继续增加吸附剂用量对萃取效果无明显影响,因此选择30 mg为最佳吸附剂用量并用于后续研究。

**图4:**
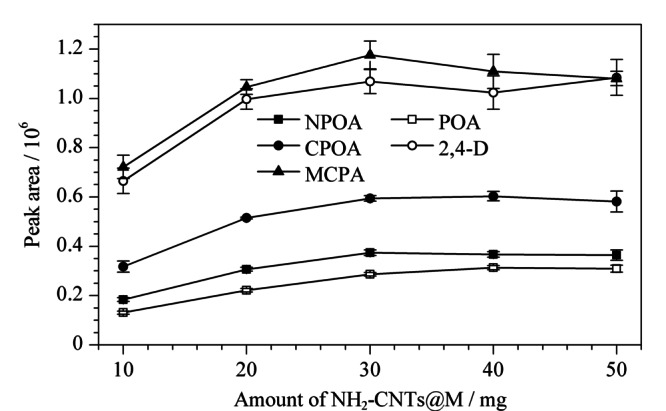
吸附剂用量对5种目标物萃取性能的影响(*n*=3)

#### 2.3.2 解吸溶剂

NH_2_-CNTs@M对PAs的萃取主要通过氢键、疏水和*π-π*等作用,因此,解吸过程中为了破坏吸附剂与PAs之间的作用力,选择含有甲酸的有机溶剂作为洗脱溶液,分别对有机溶剂的种类和甲酸的含量进行了优化。如图5a所示,与乙醇和甲醇相比,5种PAs在采用0.5 mL含1.0%(v/v) FA的ACN为洗脱液时,能达到最佳的洗脱性能,因此选择乙腈为有机萃取溶剂进行洗脱液中甲酸含量的优化。结果如图5b所示,0.5 mL含2.0%(v/v) FA的ACN可以完全洗脱NH_2_-CNTs@M吸附的PAs。根据实验结果,选择0.5 mL含2.0%(v/v) FA的ACN作为最优解吸溶剂。

**图5:**
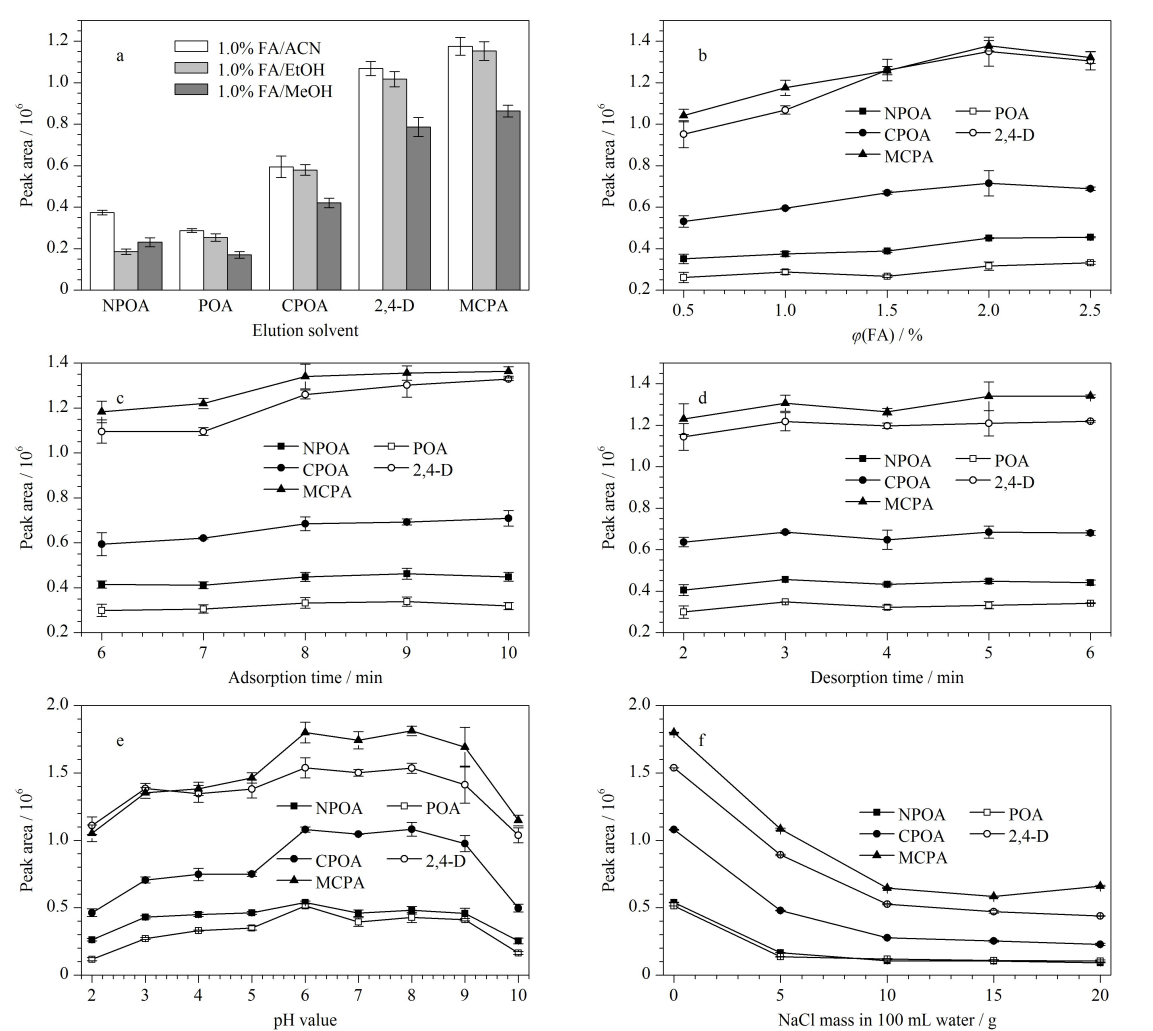
(a)解吸溶剂种类和(b)甲酸含量、(c)吸附时间、(d)解吸时间、(e)基底pH值、(f)离子强度对PAs萃取性能的影响(*n*=3)

#### 2.3.3 吸附时间

吸附时间是影响萃取性能的重要因素,足够的吸附时间可以使样品中的目标物与吸附剂充分作用,从而提高萃取效率。实验考察了不同吸附时间(6.0~10 min)对萃取性能的影响。结果如图5c所示,当吸附时间从6.0 min增加到8.0 min时,PAs的萃取性能明显增加,并在吸附时间为8.0 min时达到最佳的萃取性能,继续增加吸附时间,PAs的萃取性能变化不显著。因此,实验选择8.0 min为最优吸附时间。

#### 2.3.4 解吸时间

为了将吸附的目标物从NH_2_-CNTs@M上完全洗脱从而防止残留效应,需要足够的解吸时间。实验考察了解吸时间(2.0、3.0、4.0、5.0和6.0 min)对PAs萃取性能的影响。结果如图5d所示,当解吸时间为3.0 min时,5种PAs能够被完全洗脱。因此选择3.0 min作为后续研究的最佳解吸时间。

#### 2.3.5 溶液pH

溶液pH影响着PAs和NH_2_-CNTs@M的存在形态从而影响着吸附性能,因此选择合适的pH值可以改善萃取性能。如图5e所示,当pH值从2.0增加到6.0时,NH_2_-CNTs@M对PAs的萃取性能逐渐提高,但pH值继续提高未能进一步提高萃取效率,当pH值增加至10.0时,萃取性能明显下降。显然,在低pH时,吸附剂上的-NH_2_虽被质子化,但PAs上的羧基未发生解离,因此,NH_2_-CNTs@M主要通过*π-π*和疏水作用萃取PAs;随着pH值的升高,羧基开始发生解离,因此静电作用也参与吸附。同时吸附剂中部分氨基发生去质子化作用而导致氢键和偶极-偶极作用也参与萃取过程,在上述多重作用下,升高pH值有利于萃取。但随着pH值的继续提高,一方面纳米粒子中的氨基完全去质子化从而削弱了静电作用,同时,溶液中过量的OH^-^抑制了吸附剂与目标物之间的氢键作用,导致萃取性能下降。基于上述原因,本实验选择溶液pH值为6.0进行后续实验。

#### 2.3.6 基底离子强度

离子强度的改变可通过盐析效应和盐溶效应影响吸附剂对目标物的萃取,其中盐析效应有利于吸附剂的萃取性能,而盐溶效应则会抑制吸附剂的萃取性能^[22]^。本研究通过加入不同量的NaCl来调节样品基底的离子强度,考察了在100 mL超纯水中加入0、5.0、10、15和20 g的NaCl对NH_2_-CNTs@M/MSPE萃取PAs的影响。如图5f所示,随着NaCl加入量的增加,NH_2_-CNTs@M对目标的萃取性能显著降低,其主要是由于盐溶效应在NH_2_-CNTs@M对目标物的萃取过程中起主导作用。因此,后续实验中不调节样品离子强度。

综合以上的优化结果,本研究的最佳萃取条件为:吸附剂用量为30 mg,解吸溶剂为含2.0%(v/v)甲酸的乙腈溶液,吸附时间和解吸时间分别为8.0 min和3.0 min,基底pH值为6.0,不调节基底的离子强度。在最佳萃取条件下,NH_2_-CNTs@M/MSPE对目标物显示出理想的富集能力,对NPOA、POA、CPOA、2,4-D和MCPA的富集因子分别为79、73、89、83和90。

### 2.4 方法验证与评估

为了减少样品基底的干扰,在最佳萃取条件下,配制一系列不同浓度的样品溶液,建立基质匹配工作曲线,5种PAs在谷类和蔬菜基底中的线性范围、检出限(LOD, *S/N*=3)、定量限(LOQ, *S/N*=10)及日内日间精密度结果见表1。在大米样品中,POA的线性范围为1.0~500.0 μg/kg,其余4种目标物的线性范围均为5.0~500.0 μg/kg。在黄瓜基底中NPOA和POA的线性范围为5.0~500.0 μg/kg, CPOA、2,4-D和MCPA的线性范围为2.0~500.0 μg/kg。所有工作曲线具有良好的线性相关性(*r*^2^均大于0.99)。5种PAs在两种样品中的LOD值分别为0.32~1.6 μg/kg和0.53~1.6 μg/kg, LOQ值分别为0.94~4.8 μg/kg和1.6~4.8 μg/kg。为了评估所建方法的精密度,研究进行了10.0 μg/kg和200.0 μg/kg两种加标水平下的日内日间重复性考察。结果显示,RSD值(*n*=4)均小于10%。方法验证与评估结果表明,本研究建立的方法具有线性范围宽、灵敏度高和精密度好等优点,可用于谷类和蔬菜中PAs的分析。

**表1:** 所建立方法对PAs的分析性能

Sample	Target	Linear range^a^/(μg/kg)	r^2^	LOD/(μg/kg)	LOQ/(μg/kg)	RSDs/%(n=4)				
						Intra-day			Inter-day	
10 μg/kg	200 μg/kg	10 μg/kg	200 μg/kg
Cereal	NPOA	5.0-500.0	0.9927	1.6	4.8	9.2	6.1		8.2	7.5
	POA	1.0-500.0	0.9938	0.32	0.94	8.4	9.7		8.1	6.0
	CPOA	5.0-500.0	0.9925	1.6	4.8	5.6	3.9		6.3	3.8
	2,4-D	5.0-500.0	0.9978	1.4	4.3	8.2	5.4		6.5	6.6
	MCPA	5.0-500.0	0.9963	1.5	4.4	8.2	6.7		7.1	7.3
Vegetable	NPOA	5.0-500.0	0.9982	1.6	4.8	3.4	7.8		8.8	7.5
	POA	5.0-500.0	0.9988	1.3	3.9	4.4	9.2		7.1	9.2
	CPOA	2.0-500.0	0.9987	0.53	1.6	4.7	4.4		7.2	2.9
	2,4-D	2.0-500.0	0.9993	0.63	1.9	8.7	1.9		3.3	10
	MCPA	2.0-500.0	0.9992	0.60	1.8	4.2	1.4		8.1	1.2

MSPE: magnetic solid-phase extraction. a. Spiked levels in cereal and vegetable samples were 1.0, 2.0, 5.0, 10.0, 20.0, 50.0, 100, 200, and 500 μg/kg.

### 2.5 实际样品检测及回收率考察

为了验证所建方法的实用性,将本方法应用于谷物(大米、小米和小麦面粉)和蔬菜(黄瓜、水果黄瓜和丝瓜)样品中PAs的测定。测定结果如表2和表3所示,在一种谷物中检测到7.02 μg/kg的NPOA和5.42μg/kg的POA,在一种蔬菜中检测到5.34 μg/kg的POA和5.42 μg/kg的CPOA。为了评估样品基底的干扰,研究考察了目标物加标水平为10.0、50.0和200 μg/kg的回收率。表2和表3结果显示,谷物和蔬菜中5种PAs的加标回收率分别在73.1%~112%和72.3%~113%之间,RSD值(*n*=3)均小于10%。良好的加标回收率与实验重复性结果验证了经过NH_2_-CNTs@M/MSPE处理后,实际样品的基底对目标物的测定没有产生明显干扰。图6为大米和黄瓜样品空白及加标200 μg/kg经NH_2_-CNTs@M/MSPE富集后的谱图。

**表2:** 谷物样品中PAs的测定及加标回收率结果(n=3)

Compound	Spiked/(μg/kg)	Rice					Millet							Wheat flour				
		Found/(μg/kg)	Recovery/%	RSD/%			Found/(μg/kg)		Recovery/%		RSD/%			Found/(μg/kg)		Recovery/%		RSD/%
NPOA	0	ND				7.02							ND					
	10.0	9.33	93.3	6.3		15.9		89.2		3.0			9.57		95.7		9.7	
	50.0	38.7	77.3	8.6		48.6		107		4.8			43.1		86.1		5.3	
	200	197	98.6	5.4		178		89.1		8.4			163		81.7		5.7	
POA	0	ND				5.42							ND					
	10.0	8.38	83.8	6.1		15.3		98.9		1.7			8.49		84.9		7.4	
	50.0	56.0	112	2.1		47.4		94.8		8.2			46.2		92.3		2.5	
	200	172	85.9	7.1		199		99.6		7.2			147		73.6		4.0	
CPOA	0	ND				ND							ND					
	10.0	8.02	80.2	6.8		10.1		101		8.9			7.94		79.4		4.7	
	50.0	36.6	73.1	6.0		45.1		90.1		5.5			45.1		90.1		10	
	200	151	75.6	2.8		167		83.6		3.9			150		75.0		7.7	
2,4-D	0	ND				ND							ND					
	10.0	7.66	76.6	9.2		7.67		76.7		5.7			10.6		106		5.5	
	50.0	52.5	105	4.8		38.3		76.6		8.5			37.9		75.9		6.9	
	200	187	93.3	8.4		155		77.4		2.7			185		92.6		5.2	
MCPA	0	ND				ND							ND					
	10.0	9.94	99.4	8.9		8.09		80.9		7.8			8.81		88.1		9.3	
	50.0	42.8	85.5	9.0		41.4		82.7		9.5			39.6		79.2		8.3	
	200	169	84.7	3.3		157		78.3		7.1			163		81.6		6.2	

ND: not detected.

**表3:** 蔬菜样品中PAs的测定及加标回收率结果(*n*=3)

Compound	Spiked/(μg/kg)	Cucumber 1					Cucumber 2							Loofah				
		Found/(μg/kg)	Recovery/%	RSD/%			Found/(μg/kg)		Recovery/%		RSD/%			Found/(μg/kg)		Recovery/%		RSD/%
NPOA	0	ND				ND							ND					
	10.0	8.45	84.5	2.8		7.68		76.8		8.6			7.23		72.3		2.2	
	50.0	56.5	113	7.3		56.5		80.2		6.6			37.0		73.9		5.1	
	200	196	97.9	7.6		196		92.4		3.2			162		80.8		6.5	
POA	0	5.34				ND							ND					
	10.0	16.0	107	8.8		8.58		85.8		6.5			8.97		89.7		6.6	
	50.0	49.2	87.8	3.2		39.7		79.3		9.6			49.7		99.3		3.8	
	200	157	75.9	6.7		168		84.2		8.0			153		76.5		8.8	
CPOA	0	5.42				ND							ND					
	10.0	13.5	81.2	8.4		8.36		83.6		5.9			7.92		79.2		7.3	
	50.0	37.2	74.5	9.4		46.1		92.2		5.6			37.6		75.2		5.8	
	200	184	92.2	4.0		168		84.2		5.8			161		80.6		1.9	
2,4-D	0	ND				ND							ND					
	10.0	9.97	99.7	7.4		10.4		104		5.9			10.9		109		5.4	
	50.0	40.6	81.1	8.3		38.0		75.9		6.7			40.3		80.6		3.4	
	200	175	87.5	1.9		152		76.1		6.1			204		102		3.8	
MCPA	0	ND				ND							ND					
	10.0	10.4	104	5.9		11.2		112		9.4			10.2		102		6.6	
	50.0	38.0	75.9	6.7		40.5		80.9		5.1			42.3		84.6		8.4	
	200	152	76.1	6.1		160		79.9		5.8			176		88.0		1.0	

**图6:**
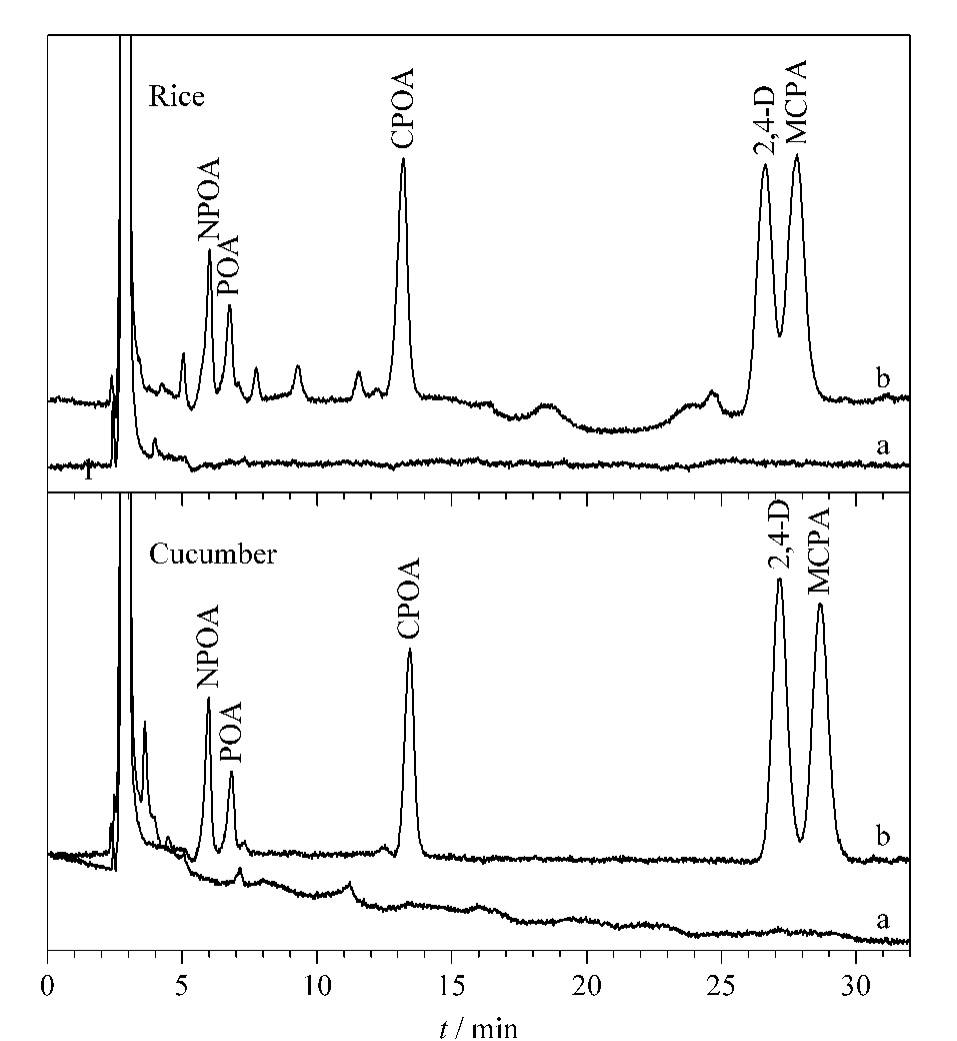
大米和黄瓜样品中PAs的色谱图

### 2.6 方法对比

为了进一步说明所建方法的特色,将本研究所建立的NH_2_-CNTs@M/MSPE-HPLC-DAD方法与已报道的用于测定谷物和蔬菜等复杂基底中PAs含量的方法进行比较,结果如表4所示。本方法与具有相同检测器的方法^[12,23]^相比具有更高的灵敏度,甚至优于HPLC-MS/MS方法取得的灵敏度^[10,24]^。同时,所建方法具有较快的萃取速度,整个萃取过程只需11.0 min,远小于其他绝大多数检测方法^[2,10⇓-12,15,23⇓-25]^。此外,相比于其他方法,本方法消耗更少的有机溶剂(仅0.49 mL)^[10⇓-12,15,25,26]^。从比较结果还可以看出,本方法取得的加标回收率与其他方法处于同一水平。

**表4:** 所建方法与文献报道的PAs检测方法的对比

Method	Sample	Compounds	Extraction time/min	Organic solvent consumption	LODs/(μg/kg)	Recoveries/%	Ref.
SPME/HPLC-UV	cereal	POA, CPOA, MCPA, NPOA, 2,4-D	80	0.39 mL MeOH	0.36-0.66	70.0-117	[2]
SPME-HPLC-UV	cereal	2,4-D	34	0.2 mL ACN	2.1	92.9-112	[23]
SPE/HPLC-MS/MS	cereal	POA, CPOA, MCPA, NPOA, 2,4-D	15	1.6 mL ACN	1.0-2.0	80.0-115	[10]
TFME/SESI-IMS	cereal	2,4-D	38	0.2 mL MeOH	0.09-0.3	82.0-115	[24]
MSPE-LLME/HPLC-MS/MS	cereal	2,4-D, MCPA, CPOA	17	4.0 mL MeOH	0.19-0.80	83.9-103	[15]
MSPE/HPLC-DAD	cereal	POA, CPOA, MCPA, NPOA, 2,4-D	11	0.49 mL ACN	0.32-1.6	73.1-112	this work
SPE/HPLC-DAD	vegetable	2,4-D, MCPA	31	0.95 mL MeOH	0.1-0.5	86.1-103	[11]
D-μ-SPE/HPLC-UV	vegetable	2,4-D	36	1.5 mL ACN	7.0	88.0-94.0	[12]
FSPE/HPLC-MS/MS	vegetable	MCPA	>25	2.85 mL MeOH	55	81.1-106	[25]
QuEChERS/HPLC-MS/MS	vegetable	MCPA	-	9.9 mL ACN	0.22-0.60	83.4-107	[26]
MSPE/HPLC-DAD	vegetable	POA, CPOA, MCPA, NPOA, 2,4-D	11	0.49 mL ACN	0.53-1.6	72.3-113	this work

TFME: thin-film microextraction; SESI-IMS: secondary electrospray ionization-ion mobility spectrometry; LLME: liquid-liquid microextraction; D-μ-SPE: dispersive micro-solid phase extraction; FSPE: filter solid-phase extraction. -: not available.

## 3 结论

本文通过“一锅法”快速制备了NH_2_-CNTs@M并将其作为MSPE的萃取介质。研究表明,所制得的NH_2_-CNTs@M由于含有丰富的功能基团,可通过多重作用实现对目标PAs的有效萃取。将NH_2_-CNTs@M/MSPE与HPLC-DAD联用,建立了谷物和蔬菜基底中PAs的分析方法并进行了实际应用研究。与现有的方法相比,所建方法具有操作简便、萃取时间短、灵敏度高和绿色环保等优点。因此本方法有望被广泛用于复杂基底中低含量PAs的常规监测。
